# Supplementary material for: Entanglement in the quantum phases of an unfrustrated Rydberg atom array
Source: Nat Commun. 2023 Sep 5;14:5397. doi: 10.1038/s41467-023-41166-0 (PMC10480489; doi:10.1038/s41467-023-41166-0)
Supplement: Supplementary file 1 — Supplementary Information [file 41467_2023_41166_MOESM1_ESM.pdf]

Supplementary Information for Entanglement in the quantum phases of an unfrustrated Rydberg atom array

Matthew J. O'Rourke[1] and Garnet Kin-Lic Chan[1]

[1]Division of Chemistry and Chemical Engineering, California Institute of Technology, Pasadena, CA 91125, USA

Supplementary Methods

This section gives additional details regarding the convergence of the numerical methods used in this work.

Γ-point DMRG: finite size errors

There are a two types of finite size errors in the energy in the Γ-point formulation of the bulk Rydberg system. These can be associated with the Rydberg interaction energy and the (emergent) kinetic energy.

As discussed in the Methods section (main text), the use of the Γ-point basis induces a periodicity in the density-density correlation function and thus in the numerator of the Rydberg interaction term. This relation is exact for classical crystals and it is also exact for quantum states with such correlations (those that can be expressed exactly in the supercell Bloch basis, which obviously need not be classical crystals). However, one can imagine that such periodic correlations are inaccurate for certain quantum phases, such as the disordered phase.

As a metric for the error *per site* induced by the constrained form of the correlations, we compute the quantity

$$\Delta e = \frac{2 \cdot R_b^6}{\rho_{ex} \cdot \min(L_x, L_y)^6} (\langle \hat{n}_i \rangle - \langle \hat{n}_i \rangle^2). \quad (1)$$

This is a measure of error for quantum crystals whose correlations do not match those induced by the Bloch basis. Note that Δe is always positive, and it can be systematically reduced by increasing the supercell size.

The other source of systematic error comes from the effective itinerancy of the Rydberg atoms arising from the $\hat{\sigma}^x$ operator [1]. The error in the kinetic energy of fermionic systems when using a Bloch basis is well studied and understood to converge superalgebraically with the supercell size $L_x \times L_y$ (see e.g. Ref. [2] and references within). We expect a similarly rapid convergence here, although the precise quantitative effect can only be directly assessed through simulations. We have carried out such checks extensively to ensure convergence of our calculations, as discussed in the following subsection and Supplementary Fig. 1.

Γ-point DMRG: convergence and physical strategy

Despite the finite size effects discussed above, we find that we can converge our calculations to sufficiently high accuracy with reasonable bond dimensions and manageable supercell sizes. Even in the very complicated region of the phase diagram near $\delta = 5.0 - 6.0, R_b = 2.3$, we can distinguish the ground-state orders using a bond dimension of $D = 1200$, as shown in Supplementary Fig. 1. However, although this is enough to identify the ground state order, higher bond dimensions would be needed to capture the phase transitions with high precision; given the large region of phase space explored here, we leave such detailed calculations to future work.

The strategy used to generate the bulk phase diagram in main text Fig. 2a, as well as the truncated interaction phase diagram Fig. 2b, is as follows.

- For a given point in phase space (δ, R_b), run a $D_{\max} = 1000$ simulation for all reasonable supercell sizes between 4×4 and 10×10, as well as 12×9.
- Identify all supercells for which the ground state has an energy per site within 10^{-2} of the lowest energy.
- If there are competing orders, ensure these solu-

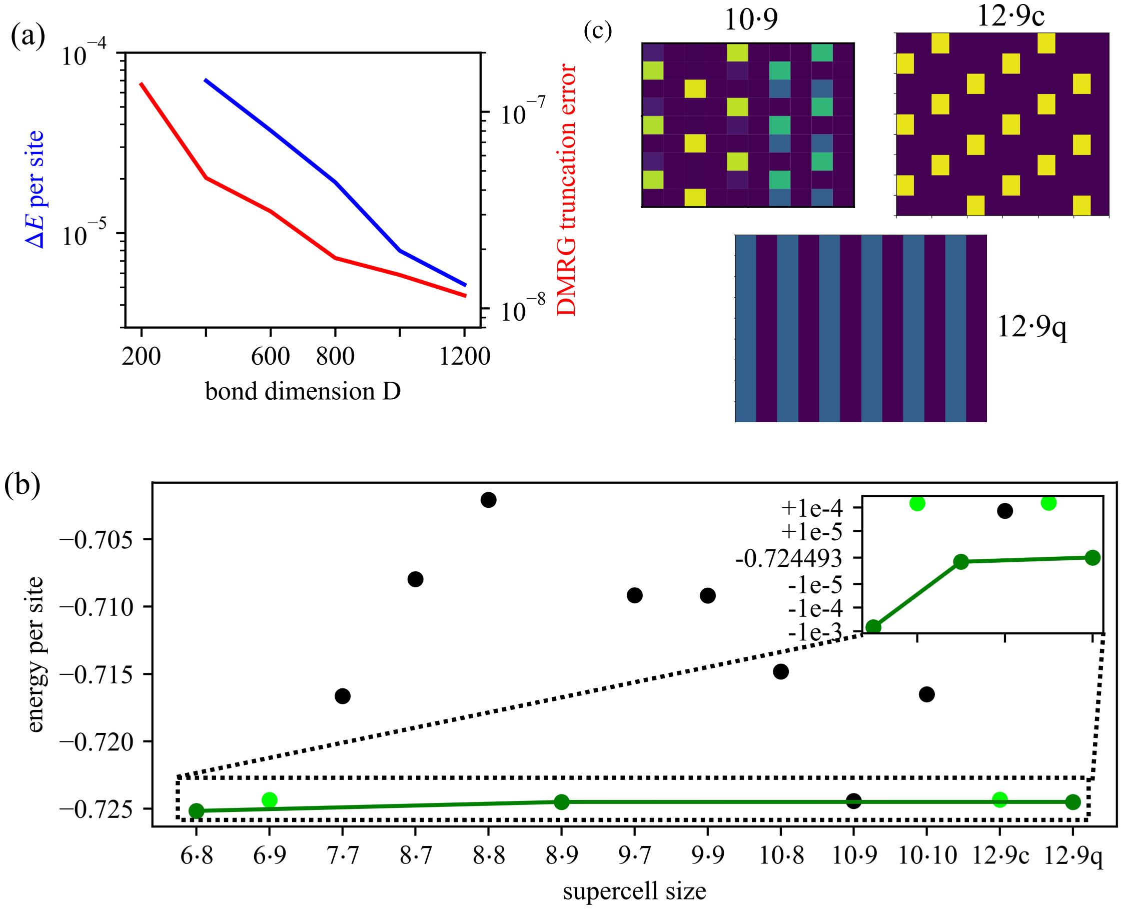

Supplementary Figure 1: Convergence of Γ-point DMRG in the most difficult region of the phase diagram $(\delta, R_b) = (5.0 - 6.0, 2.3)$. (a) Shows the convergence w.r.t. bond dimension of the largest truncated DMRG singular value (red) and the change in energy per site relative to the energy obtained with bond dimension $D - 200$ (blue). (b) The energies per site of a large variety of supercell sizes. This is adapted from Fig. 1 of the main text to highlight the relevant points. The connected dark green points are the nematic phase, and lime green points are the low energy 3-star $\frac{1}{6}$-density crystalline phase. The inset shows the convergence of the nematic phase energy w.r.t. supercell size and gaps to the other low energy solutions, whose density profiles are shown in (c). Note that, between (a) and (b), the nematic phase is converged to below 10^{-5} accuracy while the competing states differ in energy by at least 10^{-4}.

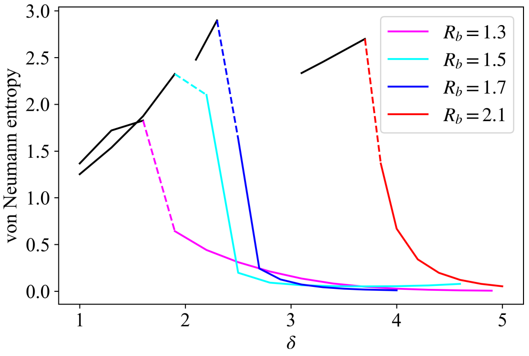

Supplementary Figure 2: Bipartite entanglement entropy of various crystalline phases as δ increases. Each line is a slice over δ values for a fixed R_b value. Black line segments denote when the ground state is in the disordered phase. Solid colored line segments denote when the ground state is an ordered crystalline phase (same color classifications as the phase diagram in main text). Dotted line segments denote the "transition zone" of a given line between the disordered phase and an ordered phase. These are simply a result of the finite resolution used to sample phase space in the phase diagrams.

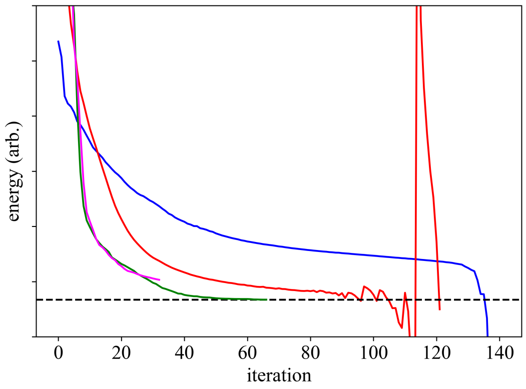

Supplementary Figure 3: Examples of typical optimization trajectories for long-range PEPS using different automatic differentiation schemes. The blue line often occurs with a naive implementation of the energy evaluation algorithms and use of a line search which does not minimize gradient norm. The red line can occur even when using a more sophisticated energy evaluation including local norms and/or a multi-evaluation cost function. The stable magenta and green lines result from combining the four techniques discussed in the Methods section (PEPS: stabilizing the optimization). The difference between the magenta and green curves reflects the quality of the initial guess.

tions are all sufficiently converged by requiring (i) the largest singular value truncated during the final DMRG sweeps is less than 10^{-8}, and (ii) corrections to the energy when increasing supercell size (up to 12×9 maximally) are smaller than the energy gap between competing states (Supplementary Fig. 1).

- The ground state phase is then identified by evaluating simple density-based order parameters on the largest supercell size which hosts the ground state order.

The only time this convergence criteria is not satisfied is for disordered phase solutions near the order-disorder phase transition (largest truncated DMRG singular value is $\sim 10^{-6}$), for which all large supercells show a disordered solution. The classification of the phase in this region is supplemented by analyzing the ground state entanglement entropy, which shows a distinctive "drop" when the phase becomes ordered (see Supplementary Fig. 2).

Importantly, this strategy completely neglects possible orders with unit cells larger than 10×10 or 12×9, as well as non-periodic solutions. Although orders with unit cells of this large size are not expected in the region of the phase diagram under investigation in this work due to the relatively high crystal densities (and thus close spatial packing) [1, 3, 4], our study cannot definitively rule out the stability of such solutions.

PEPS: convergence and physical strategy

Despite the simple procedure to generate initial guesses, we were usually able to systematically converge PEPS solutions according to the conventional protocol of increasing D and χ until the energies corresponding to multiple increasing (D, χ) pairs all vary by less than 0.01% relative to each other (e.g. see Supplementary Fig. 5). In this study, we used maximal values of $D = 5$, $\chi = 100$. However, for a small number of phase points (δ, R_b) we encountered inconsistent convergence of PEPS solutions (see Supplementary Fig. 4), where increasing D and χ did not systematically result in finding a PEPS with a lower energy, instead getting stuck in various local minima. We attribute this to the low quality of the initial guesses for larger D and R_b.

In these cases when PEPS *energies* could not be systematically converged to within 0.01%, the observed *order*

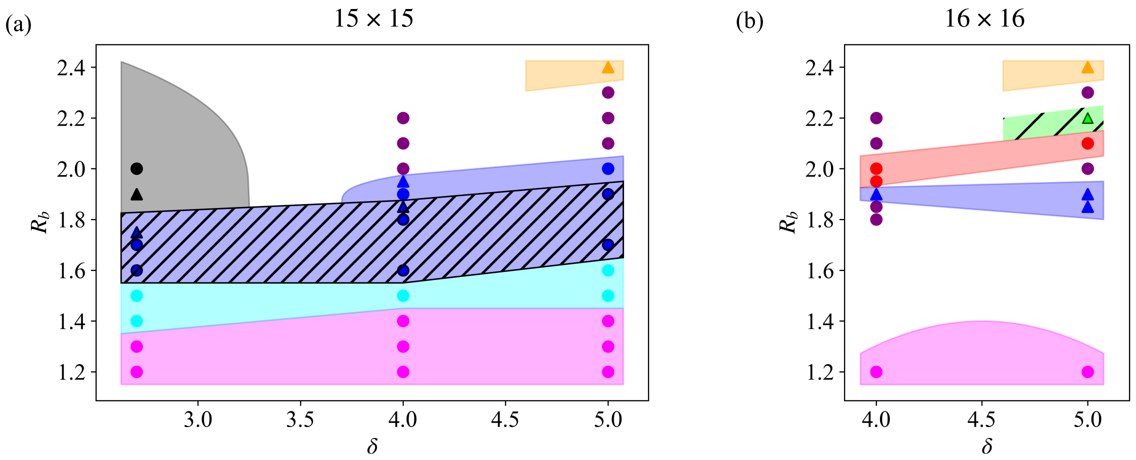

Supplementary Figure 4: Phase diagrams of 15×15 (a) and 16×16 (b) arrays, detailing convergence. Circular points indicate systematic convergence with PEPS up to bond dimension $D = 5$, while triangles indicate intermittent convergence with PEPS, requiring supplemental convergence checks using 2D DMRG. More details are available in Supplementary Note 4 and Supplementary Methods. Colors used in these plots correspond identically to the colors used in the main text to identify phases.

of the various low-energy solutions was nonetheless the same. The differing energies arose due small quantitative differences such as single-site defects and variations in the local density $\langle \hat{n}_i \rangle$. To further increase certainty in the observed order, we also compared the PEPS solutions to the results of 2D DMRG on the same finite lattice, since the convergence properties of DMRG are much more well-understood. In all cases, the low-energy PEPS solutions had similar energies to the approximate DMRG (relative difference $< 1\%$), and they all showed the same generic low-energy ground state order. The energy gap between phases appeared to be sufficiently large to allow for a tentative classification of the order of this small number of phase points, even though the DMRG was not necessarily converged to high precision (due to the wide lattices) and the PEPS convergence could not be definitively confirmed. The uncertainty in convergence highlights remaining challenges in simulating complex large 2D interacting problems with competing phases using tensor network techniques. The relevant points in the finite lattice phase diagram are labelled by triangles in Supplementary Fig. 4, and in Fig. 4 of the main text.

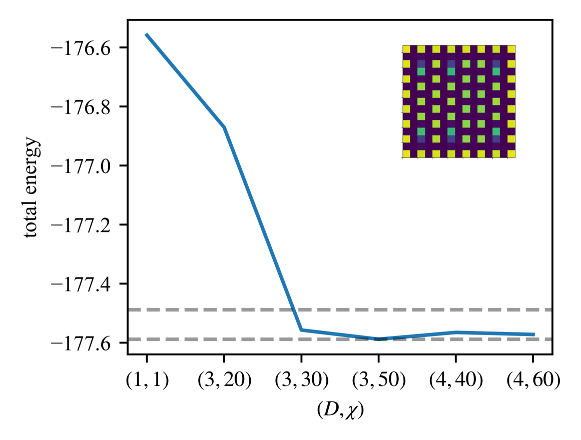

Supplementary Figure 5: An example of systematic convergence of PEPS on the 15×15 lattice for the frustrated star phase at $\delta = 4.0$, $R_b = 1.9$. The region between the horizontal lines indicates a change in energy of 0.01% relative to the lowest obtained value. The PEPS is deemed converged because many simulations with increasing (D, χ) return energies that fall within this region. Note that the star phase, like most ordered phases, is sufficiently converged by $D = 3$ due to the predominant mean-field character of most ordered phases (discussed in main text).

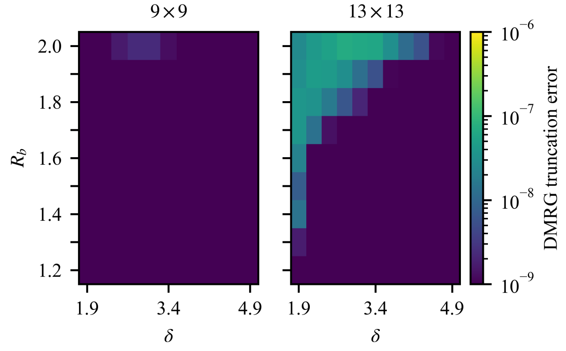

Supplementary Figure 6: Accuracy of 2D DMRG on the 9×9 and 13×13 finite lattices (open boundaries). The displayed regions of parameter space correspond exactly to the computed regions in Fig. 5 (main text) and Supplementary Fig. 14. The reported error is the largest truncated singular value during the final DMRG sweeps (i.e. once converged). Note that in the ordered regions the error is $\sim 10^{-9}$, and it grows to $\sim 10^{-7}$ as the ground state becomes disordered on the 13×13 lattice due to increasing entanglement.

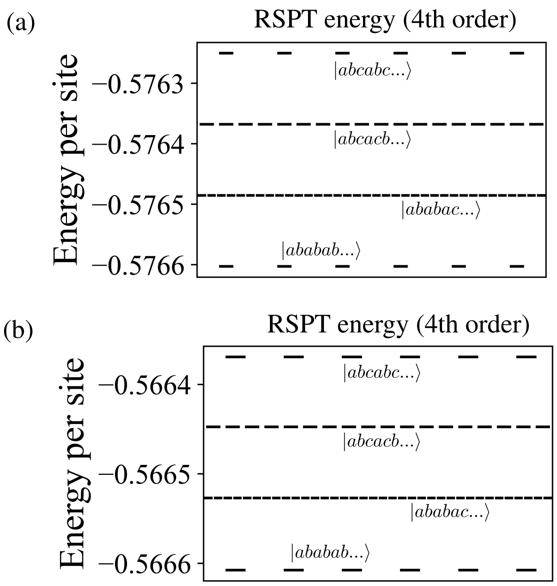

Supplementary Figure 7: Energies of various low-energy classical crystals that are corrected up to 4th-order Rayleigh-Schrodinger perturbation theory (RSPT), which includes effects of single excitation itinerancy. Results are shown for (a) interactions up to nearest neighbor columns ($|x_i - x_j| = 2$ in the 2D lattice), and (b) full long- range interactions. Comparing to the 0th-order classical energies with long-range interactions (Fig. 3d, main text), we see that the classically unfavorable states (due to longer-range terms) are stabilized by the perturbations. The energy scale of single-excitation itinerancy is therefore larger than the long-range terms.

Supplementary Note 1

Nature of the bulk nematic phase

In this section we will focus on the nature of the nematic phase and its stability in the thermodynamic limit (TDL). The qualitatively mean-field nature of all other ordered phases permits a variety of straightforward arguments for their TDL stability.

The structure of the ground state nematic wavefunction in Fig. 3 (main text) suggests that itinerancy of Rydberg excitations plays an important role in stabilizing this state. Since itinerancy of excitations and defects emerges perturbatively [1], we will rewrite the 2D Hamiltonian like,

$$\begin{aligned}\hat{H} &= \hat{H}_D + \lambda \hat{H}_Q \\ &= \frac{1}{2}\sum_{i\neq j} \frac{R_b^6}{|\vec{r}_i - \vec{r}_j|^6}\hat{n}_i\hat{n}_j - \sum_i \delta\hat{n}_i \\ &+ \lambda\sum_i \hat{\sigma}_i^x .\end{aligned}$$

Here the eigenstates of $\hat{H}_D$ are classical crystals, while $\lambda \equiv \frac{\Omega}{2} = \frac{1}{2}$.

To investigate the energy scales involved in the itinerant processes, we can begin by performing non-degenerate Rayleigh-Schrodinger perturbation theory (RSPT) starting from a *single* column state crystal taken from the quasi-degenerate exponential manifold of low energy classical states, such as $|ababab\ldots\rangle$, $|abcabc\ldots\rangle$, etc (see Fig. 3, main text). Since these initial states that diagonalize $\hat{H}_D$ are classical, with all sites having exactly $\langle \hat{n}_i \rangle = 0$ or 1, the first-order RSPT correction to the wavefunction (second-order energy) allows local superpositions of $|0_i\rangle$ and $|1_i\rangle$. This order of RSPT captures the energies of the mean-field ordered phases with high accuracy. The second-order RSPT correction to the wavefunction (4th-order energy) allows for effective "hopping" of a single excitation or defect from site i to another site j. The first- and third- order corrections to the energy are zero when starting from a single column state crystal. In Supplementary Fig. 7 we compute the energies up to the 4th order RSPT correction for various starting states, using all long-range interactions as well as interactions truncated to only be between neighboring

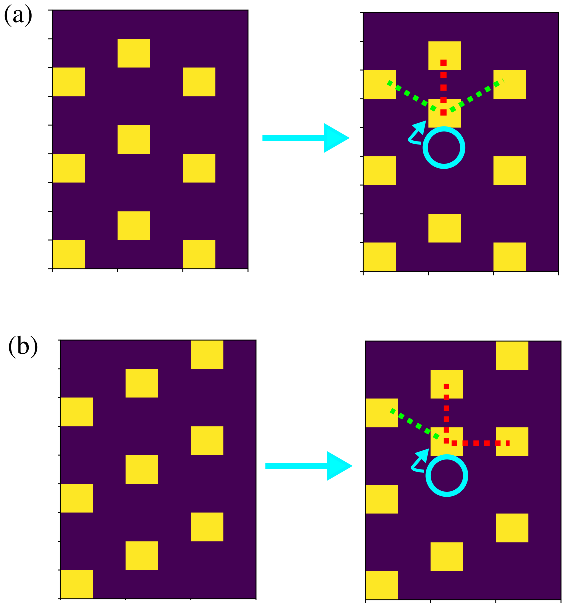

Supplementary Figure 8: Example of a perturbative hopping process for a single excitation that emerges in the second-order Rayleigh-Schrodinger correction to a crystalline initial wavefunction (fourth order energy). In (a) a single excitation in an $|ababab\rangle$-type state can hop while only violating a single Rydberg blockade constraint (denoted by a red line). In (b), a single excitation in an $|abcabc\rangle$-type state must violate two blockade constraints when hopping. These quantum fluctuations generate entanglement that preferentially stabilizes the $|ababab\rangle$-type states, despite their higher classical and mean-field energy.

columns ($|x_i - x_j| \leq 2$ on the 2D lattice; in this limit the classical energies of all low-energy column states are exactly degenerate). The states of type $|ababab...\rangle$ have the lowest RSPT energies in both interaction schemes. At the level of single-particle hopping, this is because hops within a single column from excited site y to site $y \pm 1$ can be chosen to only violate 1 Rydberg blockade ($R_b \sim 2.3$) constraint (the new excitation is 2 sites away from a single excitation within its column, but still at least $\sqrt{5}$ away from all excitations in other columns). On the other hand, states with more $|abc...\rangle$ character have some single excitation hops which must violate two instances of the blockade (they can only hop to a position at $y \pm 1$ which is 2 sites from an excitation in its own column and 2 sites from an excitation in the adjacent column). See Supplementary Fig. 8.

By comparing the results (Supplementary Fig. 7) with truncated interactions and full interactions, we can see

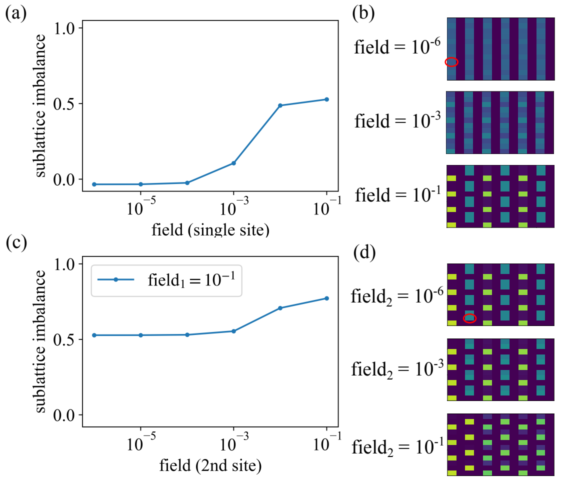

Supplementary Figure 9: Stability of the nematic order to additional single-site fields. The field is applied to only one site, and favors excitation into the Rydberg state. It can be understood as a modification of the Hamiltonian detuning term on a single site (labelled k): $-\sum_i \delta\hat{n}_i \rightarrow \sum_{i\neq k}[\delta\hat{n}_i] - (\delta + h)\hat{n}_k$. (a) Shows a measure of the "sublattice imbalance" as a function of the field h. The sublattice imbalance measures the difference in population between the preferred column state and the others ($|a\rangle$, $|b\rangle$, $|c\rangle$): $\sum_{cols}[\max(\langle\hat{n}\rangle_{|a\rangle}, \langle\hat{n}\rangle_{|b\rangle}, \langle\hat{n}\rangle_{|c\rangle}) - 0.5 \cdot \overline{\max}(\langle\hat{n}\rangle_{|a\rangle}, \langle\hat{n}\rangle_{|b\rangle}, \langle\hat{n}\rangle_{|c\rangle})]$. Here, $\langle\hat{n}\rangle_{|x\rangle}$ measures the average density of Rydberg excitations within the sublattice defined by the excitations of state $|x\rangle$, and $\overline{\max}$ is the complement of the $\max()$ operation. The sublattice imbalance is 0 in the nematic state and 1 in a pure 2D crystal. (b) Shows the ground state density profiles corresponding to representative field strengths from (a). The red circle denotes the specific site on which the field is applied. In (c)-(d), the field on the initial site is fixed to 10^{-1}, and a second field is applied to a new site in a column that has still not collapsed to a classical state.

that the energetic contributions of the quantum fluctuation induced itinerancy are larger than those of the long-range interactions, since the interaction favors $|abc\ldots\rangle$ states while itinerancy favors $|abab\ldots\rangle$ states. These fluctuations generate entanglement within the column, but also between adjacent columns because the direction of low-energy hopping is highly dependent on the state of the neighboring columns. Consequently, the large weight of the $|abab\ldots\rangle$ and related configurations in the quantum ground-state (computed with DMRG) are due to short-range entanglement. The above corresponds to an order from disorder stabilization mechanism [5], and

it is clearly stable in the TDL since it is a low-order perturbative process.

Each *single* initial crystal state for this perturbative treatment is drawn from an exponentially large, quasi-degenerate classical manifold (and there are 6 exactly degenerate permutations of the $|abab\ldots\rangle$ state). This inhibits the applicability of non-degenerate perturbation theory to provide a complete description of the true quantum ground state. A more complicated question is whether in the TDL, the short-range entangled ground-state will contain strong fluctuations around only a single non-classical configuration, or around multiple (e.g. like different Neel states in a Heisenberg antiferromagnet). At least at the level of the crystalline cell we can treat, we find the weights distributed across the 6-fold permutations of the $|abab\ldots\rangle$ configurations (as well as the exponential manifold around them). At this finite simulation size, applying a small field does not collapse the quantum state to one of the crystal states; quite a large field is required (see Supplementary Fig. 9). (By large, we mean of size 10^{-3} or larger on two sites, while the energy difference between perturbation theory (RSPT4) corrected classical configurations is on the scale of 2×10^{-4} per site. However, we note that normalising the applied field 10^{-3} on two sites, by the number of occupied sites in the classical crystals at this density, $\sim$ 24, makes the scales more comparable). Thus fluctuations and entanglement are undoubtedly much larger in this nematic phase than in the other ordered phases. However, a full picture requires a finite-size scaling analysis beyond our current calculations.

D=3 low-energy projectors and the entanglement spectrum

The character of the nematic phase has been extensively discussed in terms of the classical configurations that make up the quantum wavefunction. It has been pointed out that all the low-energy (and thus the most relevant) classical configurations can be described in a succinct notation like $|abcabc...\rangle$ in terms of compositions of 3 individual column states $|a\rangle$, $|b\rangle$, and $|c\rangle$ which are defined in Fig. 3 of the main text. This notation is very suggestive of the idea that a **qualitative** model for the 2D state can be written as a 1D MPS with a local Hilbert space of dimension 3, spanning $|a\rangle$, $|b\rangle$, and $|c\rangle$. A striking feature of the entanglement spectrum in the main text is the presence of the 3 large eigenvalues, with a $1:2$ degeneracy structure.

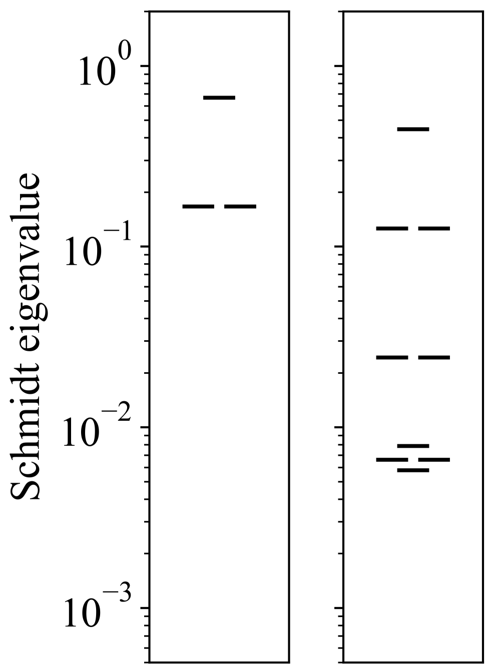

Supplementary Figure 10: Structure of the entanglement spectrum for the 1D low-energy projector model of the nematic order, as described in Supplementary Note 1 (low-energy projectors subsection). *Left*: The entanglement structure when projectors are applied on every bond of the MPS, in an open-boundary conditions style. *Right*: Entanglement structure when an additional periodic projector is applied between the first and last site in the MPS. This type of interaction is generally accounted for in the Γ-point basis calculations. Note the similarity of these Schmidt spectra to the nematic DMRG ground state shown in Fig. 3 of the main text.

To illustrate a possible origin of this pattern in the nematic phase, we create a simple model state with a similar entanglement spectrum. The strength of the interactions where the nematic phase emerges ($R_b = 2.3$) is such that configurations with adjacent columns in the same state (e.g. $|abcabb...\rangle$) are much higher in energy than all configurations without any identical adjacent columns. The projector into the low energy subspace thus removes all configurations with adjacent columns in the same state. It can be written as a product of commuting two-site operators,

$$\mathcal{P} = \prod_i \hat{P}_{i,i+1}, \tag{2}$$

where $\hat{P}_{i,i+1} = \mathbb{1} - |aa\rangle\langle aa| - |bb\rangle\langle bb| - |cc\rangle\langle cc|$.

First, we will briefly comment on the eigenspectrum of $\mathcal{P}$. Its eigenvalues are all positive integers, with the smallest being 0. The number of 0 eigenvalues grows as $\sim 2^L$, where L is the length of the 1D chain. These eigenvalues correspond to all the possible arrangements of the individual column states that do not violate any constraint in Eq. (2). This reveals the origin of the exponential classical degeneracy that has been previously discussed in the 2D system.

Concerning the entanglement spectrum, we can apply the operator $\mathcal{P}$ to a simple product state $|\psi_0\rangle$ (a $D = 1$ MPS) containing an equal mixture of all possible column states i.e. $|\psi_0\rangle = \prod_i(\lambda_a|a_i\rangle + \lambda_b|b_i\rangle + \lambda_c|c_i\rangle)$ where $|a_i\rangle$, $|b_i\rangle$, $|c_i\rangle$ represent states on column i. As long as $|\lambda_a| = |\lambda_b| = |\lambda_c|$, then $\mathcal{P}\,|\psi_0\rangle$ has the entanglement structure shown in Supplementary Fig. 10, which is very similar to that seen in the 2D nematic phase computed by DMRG.

Supplementary Note 2

Bulk phase diagram degenerate region

In the main text it was briefly mentioned that there is a small region of the bulk phase diagram where the nematic phase and 3-star phase become essentially degenerate. By this we mean that their gap becomes too small to resolve within the estimated finite size error in the Γ-point DMRG numerics. Using the Δe finite size error measure defined above, for $[R_b = 2.3, L_x = 12, L_y = 9]$, we have $\Delta e \approx 3 \cdot 10^{-5}$ in the nematic phase and $\Delta e \lessapprox 8 \cdot 10^{-6}$ in the 3-star phase. An expanded view (in δ) of the upper part of the bulk phase diagram is shown in Supplementary Fig. 11. The degenerate region emerges between the nematic phase and the $\frac{1}{5}$-staggered phase near $\delta = 7.0$, as indicated by the lime green color.

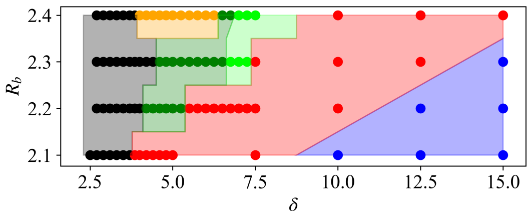

Supplementary Figure 11: Expanded view of the large-R_b part of the bulk phase diagram, computed with Γ-point DMRG. For $\delta \leq 5.0$, this data is identical to Fig. 2a in the main text. All colors correspond to the same phases as in the main text Fig. 2. The small lime green region indicates the degenerate zone where the gap between the 3-star and nematic phases becomes very small.

Supplementary Note 3

Bulk phase transitions

The disorder→order phase transitions that occur throughout the bulk phase diagram have been characterized as continuous phase transitions in previous work [3]. Although full, precise characterization of all bulk phase transitions is beyond the scope of this work, we are able to estimate the order of some transitions using straightforward numerical differentiation of the energies. Supplementary Fig. 12 shows the first and second derivatives of the energy as a function of δ, for various values of R_b, from simulations on an 8×8 supercell. The peaks in the second derivatives near the critical values of δ tentatively support the conclusions that the disorder→star and disorder→striated phase transitions are indeed second-order. However, this data is also consistent with recent work [6] which shows that although these two transitions appear continuous using an 8×8 simulation cell, they adopt strong characteristics of first-order transitions once the cell size is increased to 16×16.

Supplementary Note 4

Finite phase diagram: 15×15 and 16×16

The phase diagram of the 15×15 lattice reported in the main text contained many of the ground state orders seen in the bulk phase diagram, but it also revealed the strong finite-size effects induced by the boundary. Due to the long-range van der Waals interactions, Rydberg excitations at the edge of the array incur roughly half of the energetic penalty that excitations in the interior do, but lower the energy by an equal amount (δ). Except at small values of R_b, this induces excitations along the edge of the array to be more densely packed than what would be expected from the bulk phase diagram at a given point (δ, R_b). This generic effect causes frustration between the boundary and interior of the finite lattices, which gives rise to the square classical order and many defect-dominated states at large R_b, as discussed in the main text. In these defect states, the optimal bulk density becomes so small relative to the opti-

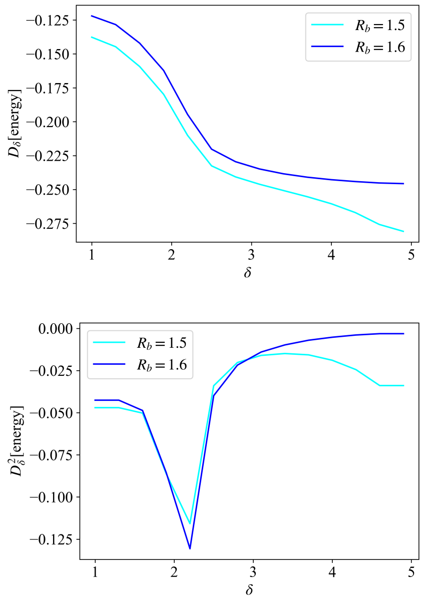

Supplementary Figure 12: Numerical evidence of second-order phase transitions between the disordered phase and the star (blue) and striated (cyan) phases, from simulations on an 8×8 supercell. *Top:* First derivative of the energy with respect to δ. *Bottom:* Second derivative of the energy with respect to δ. Both are estimated using standard finite difference formulas.

mal edge density that the ground states are permeated by edge-induced defects, leaving only small regions of any discernible order and making the precise configuration very sensitive to small changes in R_b and δ.

In addition to the 15×15 lattice, we also studied two slices ($\delta = 4.0, 5.0$) of the phase diagram of the 16×16 lattice to probe for bulk-like ordered phases where the 15×15 system is dominated by defects. Specifically, we focused on the $R_b > 1.8$ region, for which the results are shown in Supplementary Fig. 4b. We find a clear region of the stability for the boundary-bulk frustrated $\frac{1}{5}$-stagger phase (red), for which the density profile is shown in Fig. 4c of the main text. Along with a small region of the 3-star phase (green and black), these regions are unique to the 16×16 lattice (i.e. they are not seen in 15×15). There are also some common features between the two array sizes, namely regions of the star and $\frac{1}{8}$-stagger (gold) phase as well as many defect states. This suggests that the defect states are an intrinsic part of the physics of medium-sized arrays.

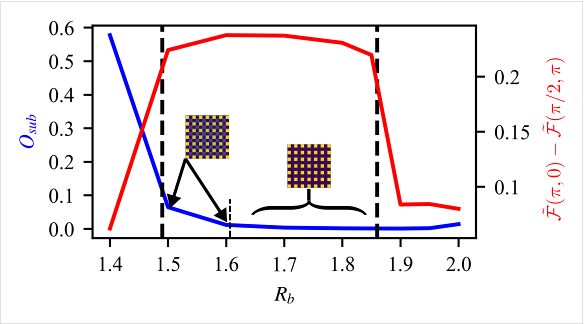

Supplementary Figure 13: Distinguishing the striated and square orders on the 13×13 lattice at the slice $\delta = 4.0$. The striated order parameter $\tilde{\mathcal{F}}(\pi, 0) - \tilde{\mathcal{F}}(\pi/2, \pi)$ (red) is large across the range $R_b = 1.5 - 1.85$, but the density of quantum fluctuations on the $(1, 1)$-sublattice O_{sub} (blue) decays to ~ 0 by $R_b = 1.65$, revealing the square order.

As reflected by the triangular markers in Supplementary Fig. 4b (which reflect inconsistent convergence) we found it more challenging than the 15×15 lattice to systematically converge the PEPS calculations with respect to (D, χ), especially in the star phase (blue). In part, this was due to the boundary itself being frustrated; on an even-sided lattice it is not possible to place excitations in all corners and also along all edges spaced by a distance of 2. Because the corner excitations are strongly pinned due to their reduced interaction penalty, this causes the boundary to be frustrated and makes it more difficult to prepare a good initial guess with our rudimentary strategies.

Supplementary Note 5

Comparing to experiment: 9×9 and 13×13 lattices

The main text discussed discrepancies between our numerical results on the 13×13 lattice and analysis reported in a recent experiment [7], specifically concerning the striated, square and star phases. It was noted that the actual experimental data appears to agree with our numerics, but the interpretation of the data offered in Ref. [7] is inconsistent with ours. This section details the effect of the approximations made in the numerics of Ref. [7] on the interpretation of the data, and how relaxing those approximation leads to the interpretation described in our main text.

Context

In Ref. [7], the experimental data on the 13×13 square lattice was primarily understood with respect to DMRG calculations performed on the 9×9 lattice (all open boundaries), in which interactions were truncated to zero beyond a distance of 2. The experimental results of Ref. [7] are reproduced in Supplementary Fig. 14a, and they are compared to our numerical results on 9×9 and 13×13 lattices (Supplementary Fig. 14b-d). The region of the phase diagram that was studied included domains of stability for the disordered, checkerboard, striated, and star phases. The square "phase" was not separately reported, although it may be considered the classical limit of the striated phase.

We also introduce here a useful order parameter for detecting the star phase,

$$O_{star} = \sum_{x,y} (\langle \hat{n}_{x,y} \rangle - \langle \hat{n}_{y,x} \rangle)^2 / N, \quad (3)$$

where $N = L_x \cdot L_y$. O_{star} detects a symmetry breaking that occurs in the star phase but not in the disordered, checkerboard, striated, or square phases. On a finite lattice, this provides a clean way to define the star phase separate from the other orders in this set. We also recapitulate the definition of the order parameters defined in [7] and used in Supplementary Fig. 14,

$$\tilde{\mathcal{F}}(k_1, k_2) = (\mathcal{F}(k_1, k_2) + \mathcal{F}(k_2, k_1))/2 \quad (4)$$

$$\mathcal{F}(k_1, k_2) = |\sum_{x,y} \exp(i(k_1 x + k_2 y)) \langle \hat{n}_{x,y} \rangle| / N. \quad (5)$$

Star phase stability

In Supplementary Fig. 14c, we recompute the main 9×9 phase diagram numerical results used in [7], which use truncated interactions. The bright region in $\tilde{\mathcal{F}}(\pi, \pi/2)$ predicts a large domain of stability for the star phase, which is corroborated by the value of O_{star}. This data was used in [7] to draw the expected phase boundary in the 13×13 experimental data seen in Supplementary Fig 14a. However, Supplementary Fig. 14d shows the analogous results on the 9×9 lattice when including all long-range interactions. Surprisingly, the star phase gets completely destabilized! This illustrates the hazard of interpreting the experimental data from smaller lattice simulations.

Unlike the 9×9 lattice, we observe that the 13×13 lattice phase diagram has a qualitative difference: it hosts a nonzero domain of star phase even when accounting for all long-range interactions. As pointed out in the main text, $\tilde{\mathcal{F}}(\pi, \pi/2)$ is not a sensitive order parameter for the star phase as it appears on finite lattices, but O_{star} does reveal the tiny stable region of the star phase (see Supplementary Fig. 14b).

Square and striated phases

The overestimation of the extent of the star phase by using numerics from the 9×9 lattice with truncated interactions also results in an underestimation of the extent of the striated order parameter, $\tilde{\mathcal{F}}(\pi, 0) - \tilde{\mathcal{F}}(\pi/2, \pi)$, since $\tilde{\mathcal{F}}(\pi/2, \pi)$ is the star order parameter used in [7] (see Supplementary Fig 14c). These 9×9 results were used in [7] to interpret the striated phase domain in the experimental data, so the boundary drawn in Supplementary Fig. 14a is too small. In fact, the extent of the experimental data for $\tilde{\mathcal{F}}(\pi, 0) - \tilde{\mathcal{F}}(\pi/2, \pi)$ (Supplementary Fig. 14a) is significantly larger than the drawn boundary, corresponding much more closely to the numerical data on the 13×13 including long-range interactions (Supplementary Fig. 14b), as mentioned in the main text.

In this work, we distinguish a region of classical square order from the striated phase where the square order contains (almost) no quantum fluctuations on the $(1, 1)$-sublattice, which are an essential feature of the striated phase in the bulk. $\tilde{\mathcal{F}}(\pi, 0) - \tilde{\mathcal{F}}(\pi/2, \pi)$ does not help distinguish between square and striated orders, and no classical square order was reported in Ref. [7]. In Supplementary Fig. 13 we show that a large part of the bright region in $\tilde{\mathcal{F}}(\pi, 0) - \tilde{\mathcal{F}}(\pi/2, \pi)$ on the 13×13 lattice should be interpreted as a classical square order by plotting,

$$O_{sub} = \begin{cases} \frac{4}{N} \sum_{x,y} \langle n_{x,y} \rangle & \text{if} x \bmod 2 = 1, y \bmod 2 = 1 \\ 0 & \text{else} \end{cases}$$

which detects the deformation of the density on the $(1, 1)$-sublattice. This sublattice is defined in terms of a 2×2 cell, as in [7].

Numerical accuracy

All numerical results in Supplementary Figs.13-14 were computed using DMRG. It was possible to study the 13×13 lattice using DMRG because we only investigated a low-entanglement region of the phase diagram. The level of accuracy for these calculations is shown in Supplementary Fig. 6 in terms of the largest truncated singular value during the DMRG sweep. In the

ordered regions of the results, the largest truncated singular value is below 10^{-9}, which is generally considered accurate.

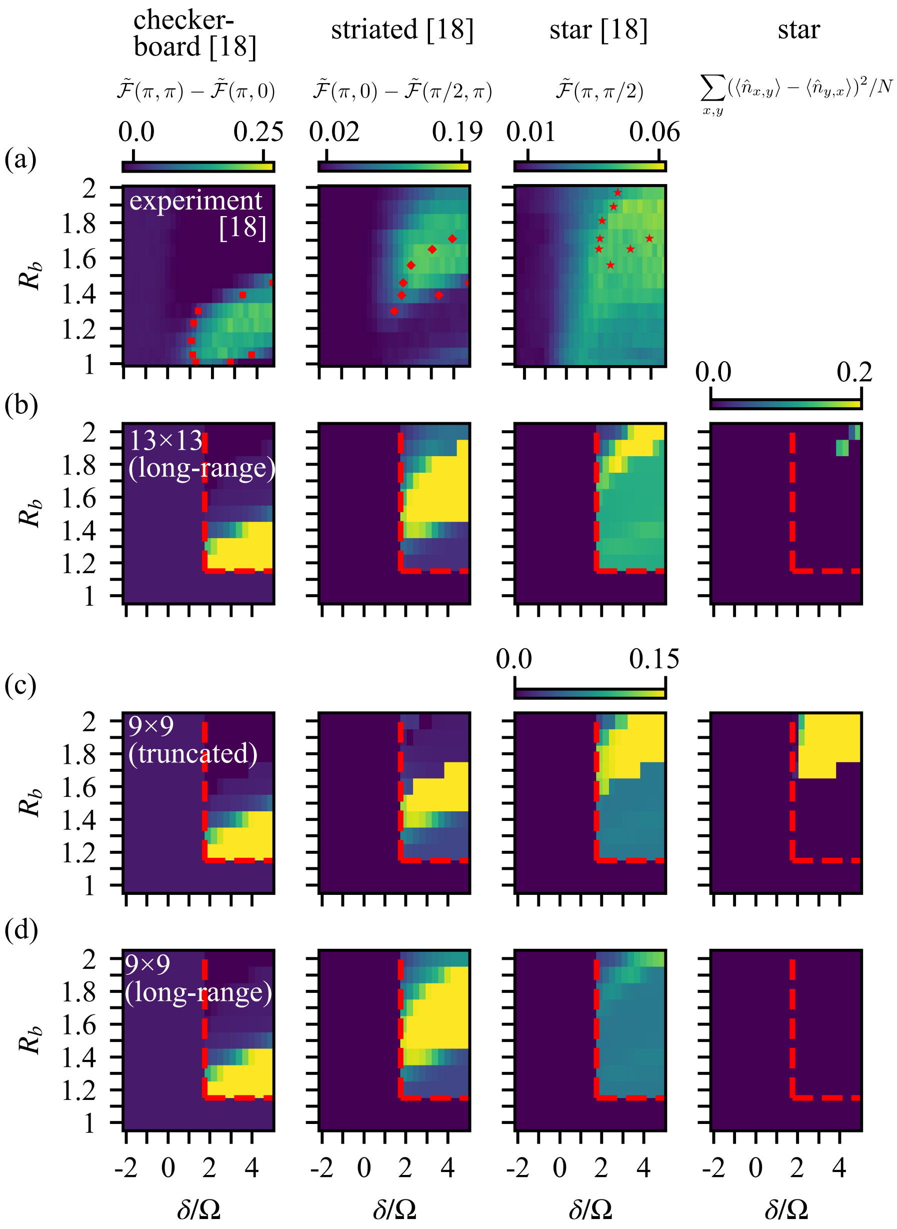

Supplementary Figure 14: Detailed comparison to experimental phase diagram. The (a) row directly reproduces the experimental phase diagram on the 13×13 lattice (data extracted from Ref. [7] Fig. 4). Rows (b)-(d) show analogous numerical data on 9×9 and 13×13 lattices, where (b) and (d) are results from simulations containing all long-range interactions and (c) shows results using interactions truncated to zero beyond distance 2. This is identical to the truncation scheme used in numerics in Ref. [7]. The first three columns show all three order parameters used in [7] to distinguish the phase diagram, while the fourth column shows a new, more precise order parameter for the star phase. Red dots in (a) denote the phase boundaries assigned in [7], while the cyan dotted lines in (b)-(d) indicate the subset of parameter space that was computed.

Supplementary References

[1] Weimer, H. & Büchler, H. P. Two-stage melting in systems of strongly interacting rydberg atoms. *Physical Review Letters* **105**, 230403 (2010).

[2] Xing, X., Li, X. & Lin, L. Unified analysis of finite-size error for periodic hartree-fock and second order m{\o}ller-plesset perturbation theory. *arXiv preprint arXiv:2108.00206* (2021).

[3] Samajdar, R., Ho, W. W., Pichler, H., Lukin, M. D. & Sachdev, S. Complex density wave orders and quantum phase transitions in a model of square-lattice rydberg atom arrays. *Physical Review Letters* **124** (2020).

[4] Rader, M. & Läuchli, A. M. Floating phases in one-dimensional rydberg ising chains. *arXiv preprint arXiv:1908.02068* (2019).

[5] Moessner, R., Sondhi, S. L. & Chandra, P. Two-dimensional periodic frustrated ising models in a transverse field. *Phys. Rev. Lett.* **84**, 4457–4460 (2000). URL https://link.aps.org/doi/10.1103/PhysRevLett.84.4457.

[6] Kalinowski, M. *et al.* Bulk and boundary quantum phase transitions in a square rydberg atom array. *arXiv preprint arXiv:2112.10790* (2021).

[7] Ebadi, S. *et al.* Quantum phases of matter on a 256-atom programmable quantum simulator. *Nature* **595**, 227–232 (2021).
